# Epilepsies and Mirror Movements: An Underrecognized Association?

**DOI:** 10.3390/jcm14082738

**Published:** 2025-04-16

**Authors:** Raffaele Nardone, Eugen Trinka

**Affiliations:** 1 Department of Neurology, Hospital of Merano (SABES-ASDAA), 39012 Merano, Italy; 2 Department of Neurology, Neurocritical Care, and Neurorehabilitation, Member of EpiCARE, Center for Cognitive Neuroscience, Christian Doppler University Hospital, Paracelsus Medical University, 5020 Salzburg, Austria; 3 Department of Neurology, “F. Tappeiner” Hospital, Via Rossini, 5, 39012 Merano, Italy; 4 Neuroscience Institute, Center for Cognitive Neuroscience, Christian Doppler University Hospital, Paracelsus Medical University, 5020 Salzburg, Austria; 5 Karl Landsteiner Institute for Clinical Neuroscience, 5020 Salzburg, Austria

**Keywords:** transcallosal pathways, interhemispheric inhibition, corpus callosum, seizures, developmental epileptic encephalopathies

## Abstract

Mirror movements (MMs) are related to structural alterations of the pyramidal tract or transcallosal pathways, as well as functional impairment of the interhemispheric inhibitory effects on motor planning skills, leading to the inability to perform limb movements independently. On the other hand, white matter bundles that connect distant cortical regions are thought to be the anatomical substrate of seizure propagation in epileptic subjects, and the spread of excitation through intracortical and transcallosal pathways is a well-recognized pathophysiological abnormality in epilepsies. To investigate this possible association, we searched the MEDLINE and Embase electronic databases, and only original articles were considered eligible for inclusion; we found thirteen patients from eleven articles, all of them case study reports. Therefore, epilepsy and MM co-morbidity has rarely been reported in the same subjects, even if changes in interhemispheric modulation are shared by both conditions. However, the study of this co-morbidity may help in elucidating the exact pathophysiological mechanisms of MMs and to better understand the pathological interhemispheric connections in epilepsy patients.

## 1. Introduction

Mirror movements (MMs) are defined as involuntary, simultaneous movements of one body side that are associated with voluntary, intentional movements on the contralateral side. They predominantly involve the distal parts of the upper limbs.

MMs are not considered a pathological finding during infancy. They usually appear before the age of five years and disappear in the first decade of life. They are most commonly due to the still immature corpus callosum (CC) [1,2] gradually disappearing and due to the maturation and myelinization of the CC and the descending tracts.

After the age of five, pathological MMs can be further distinguished between congenital and acquired MMs. Congenital MMs are often inherited autosomal dominantly and may also occur sporadically [3]. Congenital malformations are the most common cause of MMs. Acquired MMs may occur in various acquired neurological disorders [4] but also in previously healthy individuals. In humans, the analysis of grasp movement properties by means of electrocorticography data has proven to be essential for a deep understanding of the human mirror neuron system [5,6]. Interestingly, high-frequency broadband mirror and mnemonic mirror activation patterns were found in classical human mirror system sites, including the supplementary motor area and the sensorimotor, inferior frontal, and parietal cortices [5,6].

Little is known about MMs in various epilepsies and the occurrence of MMs during epileptic seizures. The communication between the brain’s hemispheres is known to be altered in persons with epilepsy, leading to an inability to suppress MMs. However, MMs in subjects with epilepsy have rarely been reported.

We aimed to review the papers reporting the occurrence of MMs in patients with epilepsy.

## 2. Methods

The MEDLINE, accessed by Pubmed (January 1966—December 2024), and Embase (January 1980—December 2024) electronic databases were checked using the medical subject headings (MeSHs) and free terms for “epilepsy”, “epileptic”, “seizures”, “mirror movements”, and “mirror activity”.

Only original articles were considered eligible for inclusion. Full-text articles were retrieved for the identified titles, and the reference lists of these articles were checked for further publications. In case data were lacking or incomplete, the authors of the studies were contacted to obtain additional information. The titles and abstracts of the initially selected articles were screened to ascertain that they satisfied the inclusion criteria. The methodological quality and risk of bias were assessed for each study. The search strategy described above yielded 11 articles, all of them case study reports; thirteen patients were reported (eight males and five females, age 6–56 years) (see Table 1).

## 3. Case Reports

Rasmussen first reported [7], in a clinical study of 17 children, adolescents and young adults without any “gross neurological deficit” and with persistent MMs. Half of the patients had neuropsychiatric deficits, and a hereditary background was found in half of the cases. Two patients had MMs and epileptic seizures [cases 4 and 16], but information about the effects of seizures on mirroring activity was lacking.

Müller et al. reported a patient with infantile left hemiparesis who underwent a hemispherectomy (HS) on the right side at age 18 for drug-resistant epilepsy [8]. Pathological examination of the removed hemisphere showed a porencephalic cyst of the temporal lobe and of the frontoparietal operculum. Movement analysis revealed normal flexion–extension synergies during walking; however, during reaching and prehension, an abnormal synergic coupling between the shoulder and elbow joint were observed. Distal limb movements could only be performed as rigidly coupled movement synergies. In the affected and healthy arm, voluntary MMs showed different phase relationships. These findings suggest that distinct circuitries are involved in the genesis of MMs and disclose the presence of a bilaterally organized cortical sensorimotor system that may enable almost normal performance of the axial–proximal body districts on both sides. It is likely that this potential cannot be otherwise exploited in subjects with only partial unilateral brain lesions and persistent motor deficits.

Takajo et al. reported a 47-year-old man with schizencephaly type II (open-lip) in the right cerebral hemisphere, suffering from focal motor and focal to generalized bilateral tonic–clonic seizures, which were well controlled with sodium valproate [9]. Clinical examination revealed MMs, mild left-sided hemiparesis, left hemiatrophy, and no sensory impairment. A plausible mechanism of the MMs was assumed to be the reorganization of the nonaffected brain and disinhibition of the ipsilateral corticospinal tract in the nonaffected left hemisphere by the transcallosal inhibitory pathway from the damaged contralateral hemisphere. Notably, sodium valproate did not reduce the MMs.

Staudt et al. reported a 6-year-old patient with a composite developmental malformation of the right hemisphere who presented with drug-resistant seizures [10]. In the neurological examination, left hemiparesis and pronounced MMs of the contralateral hand, occurring during movements of both the paretic and intact hand, were observed. Functional magnetic resonance imaging (fMRI) of repetitive unimanual grasping revealed that the two hands shared a common representation in the motor cortical region of the unaffected left hemisphere, while the undamaged right hemisphere did not show any activation during either task.

A 30-year-old right-handed woman suffering from kernicterus, deafness, slight learning disability, mild athetosis, and left temporal lobe epilepsy (with video-recorded seizures) was examined [11]. Cranial MRI revealed left hemisphere atrophy. The authors described MMs on both sides, which consisted of involuntary replication of the motor acts on the opposite side, mainly regarding the distal parts of the upper limbs. The Rasmussen clinical score of the MMs was 14 (out of 33) on the left side and 13 on the right dominant side (mean control values of 0.4 ± 0.89 and 1 ± 1.22, respectively). The maximal subscores [score 3: “MMs of the same or almost the same magnitude as the actively performed movement, or in response to passive movement”] involved pronation and supination of the forearms and wrist flexion bilaterally, sequential finger–thumb opposition, and blinded finger lifting on the left side.

Video electroencephalography (EEG) recordings in the ictal phase revealed that seizures originated from the left temporal lobe. A typical seizure was characterized initially by loss of contact and symmetric flexion of both wrists, followed by bilateral, symmetrical, and synchronous supination of the forearms. Later, a fixed dystonic posture of the right upper limb with abduction of the arm at the shoulder, flexion of the elbow, and extension of the fingers appeared. At the same time, automatisms (manipulation of the intravenous tube) occurred in the left hand. The seizure terminated quickly, without a postictal neurologic deficit. The patient was evaluated for possible surgical treatment of her severe drug-resistant epilepsy; she underwent a left temporal lobe disconnection and, afterward, remained seizure-free for 3 months.

Also, a case of polymicrogyria with benign childhood epilepsy and amyotrophic lateral sclerosis was reported [10]. The patient, a 56-year-old woman, exhibited marked unsustained MMs of the unintended hand while performing dexterity activities with either hand. Over a period of three years a moderate disease progression, with asymmetrical involvement of upper and lower motoneurons, was observed. Clinical and neurophysiological examination revealed more rapidly progressive bulbar symptoms. MRI showed polymicrogyria located in the right frontal lobe with irregular bumpy inner and outer surfaces, abnormal cortical thickening, dysplastic insular cortex, and asymmetrical widening of the Sylvial fissure. Also, atrophy, in particular, of the motor cortex and pyramidal tract, was present, while the CC was properly developed and of normal dimensions. The authors hypothesized an involvement of the dysplastic right frontal lobe in the pathophysiology of the MMs in this patient.

A 9-year-old boy, with drug-resistant focal epilepsy attributable to the same malformation of cortical development, a polymicrogyria of the right hemisphere, underwent cortical resection of the epileptic focus in the frontotemporoparietal area [13]. In the postoperative period, the patient developed left hemiplegia, and MMs were observed in the left hand. Postoperatively, he was free of seizures for four months, with residual left mild hemiparesis and persistent MMs.

The mechanisms postulated for the generation of MMs in this child included aberrant development of the corticospinal tract and transcallosal inhibitory pathways.

Sahin and colleagues also reported a patient with an enhancement in the MMs of his upper and lower limbs after epileptic seizures resulting from right frontoparietal polymicrogyria, also involving the supplementary motor area, as revealed by MRI [12]. The MMs were explored using several electrophysiological techniques, including transcranial magnetic stimulation (TMS). Since the patient manifested MMs after an epileptic seizure, the authors hypothesized that epileptic seizures increase mirroring activity by inducing cortical reorganization.

A cohort of subjects with congenital MMs in Sweden was comprehensively characterized [13]. Massive parallel sequencing evidenced two novel heterozygous frameshift variants in deleted colorectal carcinoma (DCC) gene netrin 1 receptor (*DCC*). Two siblings exhibited a complex syndrome presenting with MMs, and one case also exhibited receptive–expressive language disorder, chorea, and epilepsy (since the age of 6 years). Cranial CT showed complete agenesis of the CC (ACC) without cortical abnormalities; in particular, the anterior, posterior, and hippocampal commissures showed no abnormality. The EEG documented focal motor seizures arising in the left hemisphere with bilateral spreading during sleep, responsive to treatment with valproic acid. Clinical examination at 7 years of age revealed MMs and mild chorea, but no other motor disturbances were found. Navigated TMS demonstrated reorganized corticospinal projection patterns to the upper extremities.

Verma et al. reported a girl with progressive hemifacial atrophy and epilepsy, showing MMs in the hands [16]. The 17-year-old patient had generalized tonic–clonic seizures accompanied by loss of consciousness for the previous 12 years. In the beginning, there were four to five episodes per year, but in the last 3 years, the MMs increased in frequency up to one to two episodes daily. Headache, vomiting, arthralgia, cutaneous rash, delayed menarche, or muscle weakness were lacking in her medical history. A low hairline, a short neck, or reduced neck movements were not observed. Clinical examination revealed left face atrophy with a modest deviation in the mouth and nose to the left side. During the evaluation, when she held the examiner’s finger with her left hand, she performed analogous MMs in her right hand. No proximal upper and lower limb movements were observed. All other aspects of the neurologic exam were normal. Routine hematological and biochemical analyses were within the normal range. Brain MRI showed left cerebral atrophy, and EEG showed generalized slowing. After taking leviteracetam 1000 mg/day and oxcarbazepine 600 mg/day, the seizures were well-controlled at follow-up after 5 months.

Karatas and Saygi described two patients with septo-optic dysplasia (SOD) plus syndrome characterized by SOD and epilepsy, as well as multiple skeletal and central nervous system alterations [15], resulting from disorders in multiple developmental stages. Moreover, they first reported the occurrence of MMs in this syndrome.

Case 1 was a 46-year-old man, the first live child of a non-consanguineous marriage, with mild intellectual disabilities and motor retardation. The patient’s two older sisters had died before the age of 1 year of unknown causes, while his two younger brothers had no medical problems. After a generalized convulsive seizure at 18 months of age, a right-sided hemiparesis was noted, and thereafter, he developed focal seizures with impaired consciousness. Brain CT showed hydrocephaly on the left side. Routine EEGs revealed epileptiform discharges on the left temporal areas, sometimes spreading to the left hemisphere. Antiepileptic treatment with 300 mg/day phenytoin and 600 mg/day carbamazepine was started. The seizure frequency was 5 to 10 per year and characterized by nausea and automatism with the right hand lasting for 90 s. After 22 years of follow-up, he performed the first MRI showing septo-optic dysplasia, tetraventricular communicating hydrocephaly, partial ACC, and parenchymal atrophy of the left side of the hemisphere and brain stem.

Case 2 was a 25-year-old woman, the second born of a non-consanguineous union. A difficult delivery was reported in the patient’s medical history. During the postnatal period, intellectual and motor retardation, as well as macrocephaly (at 3 months), were observed. Brain CT revealed hydrocephaly. At the age of 18 months, she developed recurrent partial seizures with complex symptomatology, which were characterized by oral automatism, tonic posturing, and deviation of the head and eyes to the left side, persisting for 10–20 s, with a frequency once a month. Moreover, she had rare focal to bilateral tonic–clonic seizures. Antiepileptic treatment with a barbiturate was begun at 5 years of age. The family history was positive, with one of her male cousins suffering from not otherwise specified epilepsy. The left hand and foot were smaller than the right ones, and clinical examination showed left spastic hemiparesis, left homonymous hemianopia, bilateral optic atrophy, eye fixation problems, and squinting. Also, MMs of the left hand on attempted voluntary movement of the contralateral hand were observed. She was treated with 200 mg/day lamotrigine and 1200 mg/day carbamazepine. EEG revealed slow background activity, mostly on the right hemisphere, as well as sharp waves and sharp slow waves in the centroparietal region. Brain MRI showed agenesis of septum pellucidum, atrophy of the CC, and an extensive porencephalic area in the right hemisphere, with asymmetric massive dilation of the supratentorial ventricular system.

## 4. Discussion

MMs can be observed during epileptic seizures, as shown by the reported cases. The communication between the cerebral hemispheres is abnormal in epileptic subjects, leading to an incapacity to suppress MMs.

However, only a few cases have been reported of epileptic seizures with MMs, most of them suffering from childhood epilepsy due to cortical malformations.

Notably, three out of the reported thirteen patients had polymicrogyria. Polymicrogyria is a condition that affects cortical organization, defined as the thickening of gray matter, the development of multiple small gyri, producing excessive folding of the brain, and a flat border of gray and white matter [18]. Clinical findings in subjects with polymicrogyria vary, conforming to the location and extension of the lesion. The coexistence of polymicrogyria and MMs is only rarely reported. Polymicrogyria and hypoplasia of the CC were observed in cerebral MRI. According to electrophysiological studies, polymicrogyria may lead to decreased transcallosal inhibition on the affected side and enhanced activity in the ipsilateral pyramidal tract in the contralateral hemisphere, thus determining the onset of MMs [13].

Rasmussen reported that pronation and supination of the forearm were the most evidently mirrored movements, either interictally or during an epileptic attack [7]. A possible role of the contralateral nonepileptic motor cortex in the occurrence of MMs seems to be unlikely since ictal motor behavior was associated with restricted and well-lateralized discharge without generalization [11].

The spread to the contralateral hemisphere of focal epileptic activity demonstrated in this patient with ACC is interesting because the CC represents a crucial pathway for bilateral synchrony [19], indicating propagation through noncanonical routes [11].

Navigated TMS demonstrated a reorganization of corticospinal projection patterns to the upper extremities.

Early hemispheric lesions may determine a hypertrophy of the contralateral corticospinal tract containing more ipsilateral fibers [10]. In the study by Staudt and co-workers, the patient exhibited a congenital cortical abnormality on the right side, which could result in ipsilateral corticospinal projections on the contralateral side, but fMRI failed to support this hypothesis.

An analogous case of MMs, together with focal onset seizures, was reported by Takajo et al. in a subject with open-lip schizencephaly [9]. A neuronal reorganization and reallocation of motor control on the nonaffected cerebral hemisphere was postulated. The lack of transcallosal inhibitory control from the damaged hemisphere may produce an overflow of the stimulatory inputs from the nonaffected hemisphere, thus leading to the simultaneous movement of homologous muscles.

In the patient described by Verma et al., the MMs were probably related to cerebral hemiatrophy [16]. A possible explanation for this association was hypothesized to be insufficient transcallosal connections, enhanced activation of the nonaffected cerebral cortex, or the development of aberrant corticospinal connections.

This assumption is supported by the finding of acquired MMs in a case of drug-resistant epilepsy following focal cortical resection due to polymicrogyria [13]. In this patient, the surgical resection resulted in left hemiplegia and in a failure of the right hemispheric transcallosal suppression of the ipsilateral corticospinal projections from the contralateral hemisphere, causing MMs in the paretic hand on voluntary movements of the healthy hand. Functional ipsilateral pyramidal projections from the left hemisphere were likely to exist even before surgery because of the known congenital malformation resulting in reduced transcallosal inhibition from the damaged hemisphere. However, MMs were not evident before surgery, and no activation of the healthy hemisphere on voluntary motor activity of the left hand during fMRI was detected. A possible reason for the absence of presurgical MMs and the occurrence of postresection MMs is that the affected cortex may be able to provide effective transcallosal inhibition. On the other hand, continuous epileptic activity originating from the right motor cortex might have been adequate to preserve the tonic transcallosal inhibition coming from the right hemisphere.

Notably, white matter bundles that connect distant cortical regions also are believed to be the anatomical substrate for seizure propagation [20] and, among them, the CC constitutes the largest commissure connecting the two hemispheres [21]. Cortical excitability changes in both hemispheres, ipsilateral and contralateral to the epileptic focus (i.e., “focal” and “non-focal” hemispheres, respectively), may represent a basic mechanism for the propagation of epileptic discharge and may differentiate focal from generalized epilepsies [22]. Another factor may be an amplified interhemispheric transmission or defective inhibition through the CC.

Indeed, the CC facilitates the propagation of epileptic activity in animal models [23,24,25], as well as in subjects with severe drug-resistant epilepsies, showing a significant decrease in generalized seizures after palliative callosotomy [26]. Therefore, an impaired inhibitory response of contralateral M1 to signals traveling from the hemisphere with seizure focus might represent a background factor for the spread of focal discharges in the contralateral hemisphere and, thus, for the evolution into generalized seizures.

The predisposition for the spread of excitation through callosal and intracortical pathways is also a well-recognized pathophysiological alteration in subjects with cortical myoclonus, and it may play an important role in the generalization of the seizures observed in these patients [27].

In conclusion, changes in interhemispheric modulation are shared by subjects with epilepsy and MMs. This association may be, therefore, of great scientific and clinical interest.

However, it should be noted that all data came from isolated case reports, with a high risk of bias and limited generalizability. Another limitation of this narrative review is that the exact pathophysiological mechanisms of MMs were not elucidated, and, therefore, the possible explanations of the link between MMs and epilepsies remain speculative.

To address the current gaps, future research should include prospective observational studies, standardized neurophysiological assessments, and molecular genetic testing. Neuroimaging methods, such as diffusion tensor imaging and fMRI, as well as neurophysiological techniques, in particular, electroencephalography with simultaneous electromyography and transcranial magnetic stimulation, may help to investigate this co-morbidity. Moreover, their combined use should be encouraged.

Future studies might shed light on the propagation mechanisms via interhemispheric connections and on the pathological interhemispheric communications in epilepsy patients. Elucidating these underlying mechanisms could allow the development of new diagnostic and therapeutic approaches in epilepsies.

## Figures and Tables

**Table 1:** Demographic, clinical, and neuroimaging characteristics of the patients.

Studies	Nr	A/G	Epilepsies	Other Clinical Features	Neuroimaging
Rasmussen et al., 1993 [7]	2	15/M	Focal seizures, complex symptomatology	Mild DAMP	Not reported
		14/M	Focal seizures with impaired consciousness	Tics	Not reported
Müller et al., 1990 [8]	1	50/F	Generalized and focal seizures	Hemiampia to the L	CT: porencephalic cyst of the R temporal lobe
				Sensorimotor deficits L	and of the R frontoparietal operculum
Takajo et al., 2000 [9]	1	47/M	Focal motor seizures to bilateral tonic–clonic seizures	Mild L hemiparesis, L hemiatrophy	MRI: schizencephaly
Staudt et al., 2001 [10]	1	6/M	Drug-resistant epilepsy with many different seizure types	Spastic L hemiparesis	MRI: complex malformation of R hemisphere
Vercueil et al., 2002 [11]	1	30/F	L temporal lobe epilepsy	Kernicterus, deafness, mental retardation athetosis	MRI: L cerebral atrophy
Krampfl et al., 2003 [12]	1	56/F	Benign childhood epilepsy	Amyotrophic lateral sclerosis	MRI: L frontal polymicrogyria
RamachandranNair et al., 2006 [13]	1	9/M	Focal seizures to bilateral tonic–clonic seizures	Mild weakness L upper limb	MRI: malformation of cortical development
					frontal, parietal, and temporal R
Sahin et al., 2006 [14]	1	28/M	Focal seizures to bilateral tonic–clonic seizures	Increased deep tendon reflexes and Babinski on the L side	MRI: R frontoparietal polymicrogyria
Karatas and Saygi, 2009 [15]	2	46/M	Nausea and right-hand automatism	Dysarthria, bilateral optic atrophy	CT: septo-optic dysplasia, tetraventricular hydrocephaly,
				R facial paralysis, R spastic hemiparesis	partial agenesis of the CC, atrophy of the L hemisphere and brain stem
		25/F	Focal seizures with automatisms and tonic posturing	R hemihypoesthesia, L spastic hemiparesis, fixation problem, bilateral optic atrophy, L hemianopia	CT: agenesis of septum pellucidum, atrophy of CC, porencephaly R hemisphere, asymmetric hydrocephaly
Verma et al., 2015 [16]	1	17/F	Bilateral tonic–clonic seizures	Atrophy of L face	MRI: L cerebral atrophy
Thams et al., 2020 [17]	1	7/M	Not reported	Receptive–expressive language disorder	CT: complete agenesis of the CC
				chorea	

Legend: Nr = number of patients; A = age; G = gender; M = male; F = female; L = left; R = right; CT = computed tomography; MRI = magnetic resonance imaging; DAMP = deficit in attention, motor control, and perception; CC = corpus callosum.
